# Inhibition of integrin β3, a binding partner of kallistatin, leads to reduced viability, invasion and proliferation in NCI-H446 cells

**DOI:** 10.1186/s12935-016-0365-7

**Published:** 2016-12-01

**Authors:** Guoquan Wang, Xiao Wang, Xiaoping Huang, Huiyong Yang, Suqiu Pang, Xiaolan Xie, Shulan Zeng, Junsheng Lin, Yong Diao

**Affiliations:** 1 Institute of Molecular Medicine, Huaqiao University, Quanzhou, 362021 China; 2 College of Chemical Engineering and Materials Sciences, Quanzhou Normal University, Quanzhou, 326000 China; 3 School of Chemistry and Chemical Engineering of Guangxi Normal University, Guilin, 541004 China

## Abstract

**Background:**

Kallistatin is a serine proteinase inhibitor and heparin-binding protein. It is considered an endogenous angiogenic inhibitor. In addition, multiple studies demonstrated that kallistatin directly inhibits cancer cell growth. However, the molecular mechanisms underlying these effects remain unclear.

**Methods:**

Pull-down, immunoprecipitation, and immunoblotting were used for binding experiments. To elucidate the mechanisms, integrin β3 knockdown (siRNA) or blockage (antibody treatment) on the cell surface of small the cell lung cancer NCI-H446 cell line was used.

**Results:**

Interestingly, kallistatin was capable of binding integrin β3 on the cell surface of NCI-H446 cells. Meanwhile, integrin β3 knockdown or blockage resulted in loss of antitumor activities induced by kallistatin. Furthermore, kallistatin suppressed tyrosine phosphorylation of integrin β3 and its downstream signaling pathways, including FAK/-Src, AKT and Erk/MAPK. Viability, proliferation and migration of NCI-H446 cells were inhibited by kallistatin, with Bcl-2 and Grb2 downregulation, and Bax, cleaved caspase-9 and caspase 3 upregulation.

**Conclusions:**

These findings reveal a novel role for kallistatin in preventing small cell lung cancer growth and mobility, by direct interaction with integrin β3, leading to blockade of the related signaling pathway.

## Background

Integrins are transmembrane receptors composed of two subunits, α and β chains. They comprise a large family of cell surface receptors, with more than 18 α subunit and 8 β subunit isoforms identified in mammals. Integrins play a role in bridging cell–cell and cell-extracellular matrix (ECM) interactions [1, 2]. Increasing evidence indicates that the integrin family initiates intracellular signaling events that promote tumor cell proliferation, survival and migration. The signaling events stimulated by integrin members are transduced into the cell via activation of integrin-associated proteins such as Src-family protein-tyrosine kinases (PTKs), including focal adhesion kinase (FAK) [3, 4]. Most integrins recruit FAK through their β subunits. Integrin β3, a cell surface adhesion molecule, is largely considered a driver of tumor progression [5]. Based on integrin β3 location on the cell surface of small cell lung cancer cells, and its role in promoting anchorage-independent survival [6, 7], it is reasonable to consider integrin β3 a potential inducer of tumor cell survival during invasion and metastasis while facing environmental changes. Tyrosine phosphorylation of integrin β3 leads to conformational change and activated form that facilitates FAK activation through auto phosphorylation at Y397. This phosphorylation creates a high-affinity binding site for Src, and subsequently the FAK-Src signaling complex. Activated Akt and Erk1/2 then perform various cell survival functions, generally resulting in upregulated Bcl-2 expression and downregulated pro-apoptotic molecules [8–10]. As an inducer of the Ras/MEK/ERK pathway, growth factor receptor-bound protein 2 (Grb2) is crucial for regulating cell proliferation and tumorigenesis. Grb2 is a key adaptor protein in maintaining ERK activity by linking Son of sevenless homolog (Sos) or other proteins to activated RTKs, such as EGFR. Upon activation of EGFR or other RTKs, Grb2 recruits Sos1 to the membrane to form the Grb2-Sos complex, which is crucial for signal transduction, sequentially leading to Ras/MEK/ERK activation [11–14].

Kallistatin is a serine proteinase inhibitor and heparin-binding protein. It plays multiple biological roles, including inhibition of angiogenesis, inflammation, tumor growth, and metastasis, as demonstrated in a number of animal models and cultured cell lines [15–18]. Kruppel-like factor 4 (KLF-4) was shown to mediate the anti-inflammatory action of kallistatin by increasing endothelial nitric oxide synthase (eNOS) expression in endothelial cells [19]. More recently, kallistatin was shown to inhibit cancer cells directly [20–22]. Indeed, kallistatin could bind to the Wnt co-receptor low-density lipoprotein receptor-related protein 6 (LRP6), thus blocking Wnt/b-catenin signaling as well as Wnt-mediated growth and migration in MDA-MB-231 breast cancer cells [20]. We recently demonstrated that kallistatin inhibits proliferation of lung cancer cells and enhances apoptosis in vitro, therefore inhibiting lung cancer in a subcutaneous NCI-H446 xenograft model by reducing tumor cell angiogenesis and proliferation [23]. However, little is known about the molecular mechanisms by which kallistatin reduces lung cancer cell viability, proliferation and migration, in particular through the integrin signaling pathway.

The present study aimed to identify specific kallistatin binding protein(s) or kallistatin receptor(s) on the cell surface for understanding the molecular mechanisms by which kallistatin inhibits NCI-H446 cell viability, proliferation and migration.

## Methods

### Reagents

His-tag recombinant human kallistatin was expressed in *Pichia pastoris* strain GS115 and purified by a series of chromatographic steps, mainly Phenyl Superose and Heparin Sepharose FF chromatography [24, 25]. Rabbit anti-human integrin β3, rabbit anti-human phospho-Integrin β3, and mouse anti-human integrin β3 were purchased from Santa Cruz Biotechnology (Santa Cruz, CA). Rabbit anti-human AKT, rabbit anti-human phospho-AKT, rabbit anti-human Erk1/2, rabbit anti-human phospho-Erk1/2, rabbit anti-human FAK, rabbit anti-human phospho-FAK, rabbit anti-human Src and rabbit anti-human phospho-Src were obtained from Cell Signaling Technology (Boston, MA). 3-(4,5-dimethylthiazol-2-thiazolyl)-2,5-diphenyl-tetrazolium bromide was purchased from Sigma. Click-iT EdU Alexa Fluor 594 Imaging Kit (including Hoechst 33,342 and Apollo reaction cocktail) and Lipofectamine™ 2000 were from Life Technologies (Gaithersburg, MD). PVDF membranes and the Immobilon ECL detection system were purchased from Millipore Co (Billerica, MA). Integrin β3 and negative control siRNA were purchased from GenePharma (Shanghai, China).

### Cell viability assay

Cell viability was assessed by MTT (3-(4,5-dimethylthiazol-2-yl)-2,5-diphenyl-tetrazolium bromide) assay. Chinese hamster ovary (CHO) (ATCC, PTA-3275) and NCI-H446 cells (ATCC, HTB-171) were obtained from American Type Culture Collection. CHO, as a non-integrin β3–expression cell line, was used as a control. NCI-H446 cells were cultured in RPMI 1640 medium supplemented with 10% fetal bovine serum (FBS), penicillin (100 U/ml) and streptomycin (100 μg/ml) in a humidified atmosphere of 5% CO_2_ at 37 °C. CHO cells were cultured in F-12 Kaighn’s modification medium supplemented with 10% fetal bovine serum. Cells (3 × 10^3^) were divided into seven groups (six wells per group) and incubated in 96-well plates with various concentrations of kallistatin (0, 6.25, 12.5, 25, 50, 100, and 200 μg/ml), respectively. After 48 h of incubation, the cells were treated with MTT (50 μg/well) for additional 3 h. The supernatants were discarded before addition of 200 μl/well DMSO for 10 min with vigorous shaking. Finally, absorbance was read on automatic plate reader (molecular devices) at 490 nm, with 630 nm used for background correction. The experiments were repeated three times independently.

### EdU proliferation assay

Proliferation of NCI-H446 cells was assessed by using Click-iTEdU Alexa Fluor 594 Imaging Kit according to the manufacturer’s instructions. Briefly, cells were pre-treated with kallistatin or PBS for 48 h, and incubated with 10 μM EdU for 4 h at 37 °C. Cells were then fixed with 4% formaldehyde for 15 min, and treated with 0.5% Triton X-100 for 20 min at room temperature for permeabilization. After three washes with PBS, cells were incubated with the Apollo reaction cocktail for 30 min. DNA was stained with 10 μg/ml of Hoechst 33,342 stain for 20 min and visualized by fluorescence microscopy. More than five random fields per well were captured at 100× magnification, and IPP 6.0 was used to calculate the percentage of EdU-positive cells (identified by Apollo® 594 fluorescence) in total cells (identified by Hoechst 33,342 nuclei staining). In some experiments, cells were pre-treated with siRNA or antibody for 24 h prior to kallistatin treatment.

### Cell migration assay

Migration assay was performed using Transwell (Costar, NY, USA; pore size, 8 μm) in 24-well dishes. NCI-H446 cells were harvested and resuspended at a density of 10^4^ cells/0.2 ml in serum-free medium. The bottom chamber was filled with 0.3 ml of the corresponding medium, containing 12.5, 50 and 200 μg/ml kallistatin, respectively. The upper chamber was loaded with 0.1 ml medium containing the cells and 12.5, 50, and 200 μg/ml kallistatin, respectively, and incubated for 12 h at 37 °C, with 5% CO_2_. The cells were fixed in 20% methanol for 15 min and stained with 0.1% crystal violet in PBS (v/v) for 15 min. Cells on the upper side of the filters were removed with cotton tipped swabs, and the filters washed with PBS. Cells on migrated through the filters were examined and counted under a microscope. Experiments were carried in triplicate, and repeated at least three times.

### siRNA transfection

NCI-H446 cells were seeded into 6-well tissue culture plates without antibiotics. After 24 h, cells reached 70–80% confluence, and were washed twice with PBS. The double-strand siRNA was 5-CGGUGAGCUUUAGUAUCGATT-3 for integrin β3. Scrambled siRNA, which served as negative control, was purchased from GenePharma (Shanghai, China). Double-stranded negative control (NC) siRNA was used in parallel. The cells were then transfected with double-stranded small interfering RNA (15 nmol/l) using Lipofectamine™ 2000 (5 μl/well) in Opti-MEM medium, following the manufacturer’s instructions [26]. Cells were incubated for 24 h before further experiments.

### His pull-down assay

His-tag recombinant human kallistatin was expressed in *P. pastoris* strain GS115 and purified by chromatographic steps, Phenyl Superose and Heparin Sepharose FF chromatography [24]. Sub confluent NCI-H446 cells were collected with EDTA and washed twice with cold PBS. The cells were resuspended in 1 ml ice-cold hypotonic buffer [10 mM HEPES, pH 7.9, 0.5 mM dithiothreitol, 0.5 mM phenylmethylsulfonyl fluoride, and a protease inhibitor mixture tablet (Roche, Milan, Italy)] [27]. Then, cells were disrupted with 50 strokes of a tight-fitting Dounce homogenizer (Beytime, Shanghai, China). The resulting homogenate was assessed under a NikonTX100 phase contrast microscope (Tokyo, Japan) to confirm that no intact cell remained. After centrifugation of the homogenate to remove nuclei and mitochondria at 8000*g* for 10 min, the supernatant was subjected to another centrifugation at 100,000*g* for 30 min. The membrane fraction, obtained as the pellet, was dissolved in 200 μl hypotonic buffer. Membrane proteins were released by treatment with 1% Triton X-100 for 1 h, and samples (10 mg) were incubated with 1 ml kallistatin-Ni–NTA affinity beads for 16 h at 4 °C. Same amount of empty Ni–NTA beads were used as a control. The reactions were loaded into column, respectively. Unbound proteins were removed by washing with PBS. The fractions were collected when the complex of kallistatin-target membrane protein was eluted from the column. 40 µl from each fraction were applied to reducing sodium dodecyl sulfate–polyacrylamide gel electrophoresis (SDS-PAGE, 12%), followed by immunoblotting using anti-kallistatin primary antibody.

### Immunoprecipitation

Immunoprecipitation of the kallistatin complex with its binding protein was performed as previously described [28]. The whole-cell lysate of NCI-H446 cells was pretreated with protein A-Sepharose beads for 1 h at 4 °C with rotation. Protein A-Sepharose was pelleted by 1500*g* centrifugation to remove potentially immunoglobulin contaminants from the original samples. The supernatant was transferred into a flesh microcentrifuge tube followed by incubation with either rabbit immunoglobulin (as control) or mouse anti-kallistatin antibody with shaking at 4 °C overnight. Protein A beads were then added with continuous shaking for another 2 h. The samples were then centrifuged, and the resulting pellets were washed 4 times with PBS and subjected to SDS-PAGE and immunoblotting.

### Immunoblotting

NCI-H446 cells were plated at 6 × 10^5^ in 6 cm dishes, and treated with kallistatin for 24 or 48 h. Cells were lysed in whole cell lysis buffer (50 mM Tris, pH 7.5, 1% NP40, 150 mM NaCl, 10% glycerol, 1 mM EDTA) supplemented with protease and phosphatase inhibitor cocktail tablets (Amersco). Protein samples either prepared by whole cell lysis, His pull-down or immunoprecipitation were boiled for 10 min before separation by SDS/PAGE (8–12% gels) and subsequent transfer onto PVDF membranes (Millipore). The membranes were incubated with designated primary antibodies against kallistatin, integrin β3, phospho-integrin β3, AKT, phospho-AKT, Erk1/2, phospho-Erk1/2, Src phospho-Src, FAK and phospho-FAK, respectively. After washing of unbound primary antibodies, the membranes were incubated with appropriate HRP conjugated secondary antibodies, respectively. Immunoreactive protein bands were visualized by the enhanced chemiluminescence detection system and photographed on a Molecular Imager Gel Doc XR system (Bio-Rad). Intensity was measured with the Image J software assess relative protein levels.

### Statistical analysis

Data are mean ± SD. Comparisons among groups were performed by one-way analysis of variance (ANOVA), followed by Scheffe’s test. *P* < 0.05 was considered statistically significant.

## Results

### Integrin β3 is a kallistatin binding protein

**Fig. 1:**
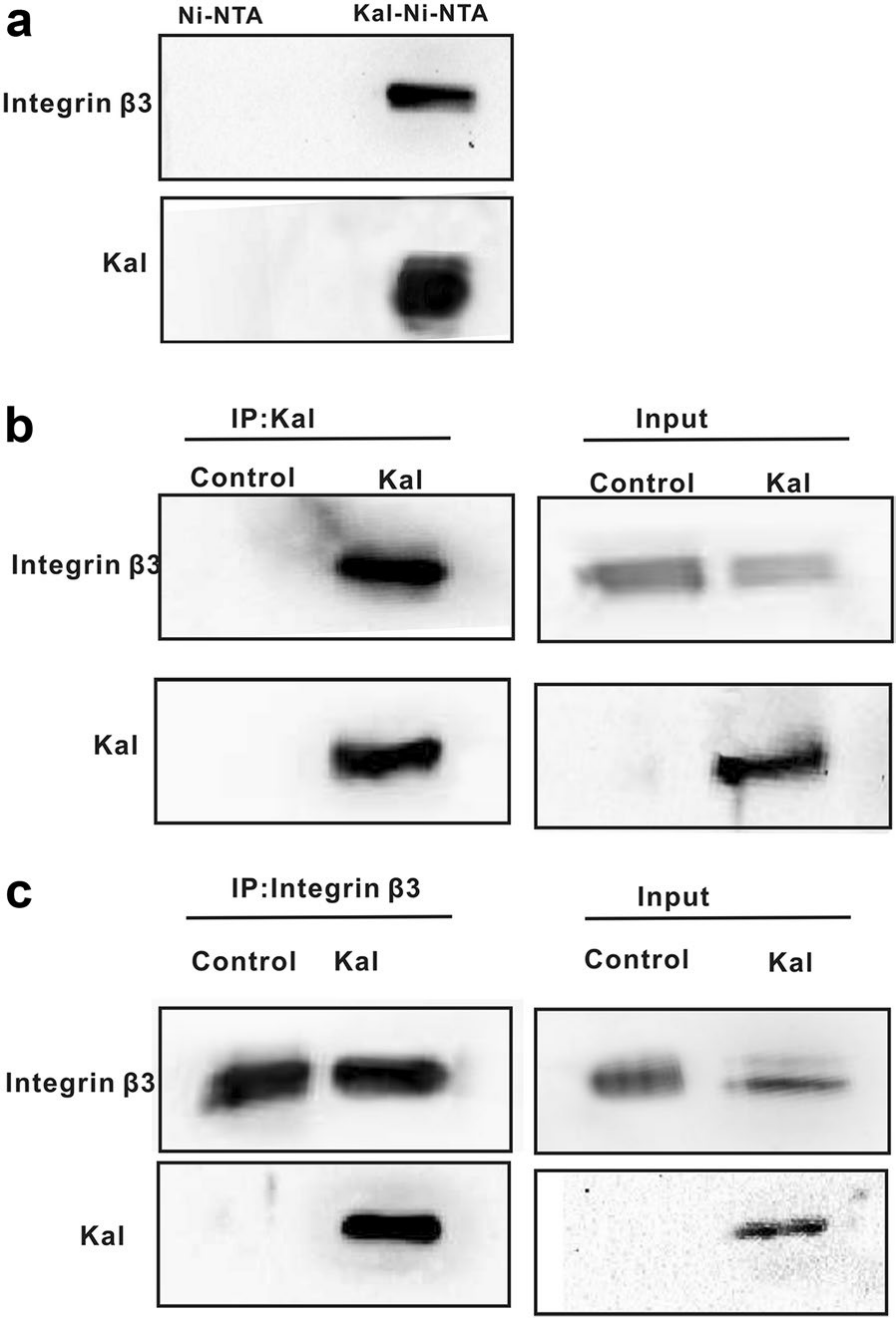
Identification of integrin β3 as a kallistatin-binding protein. **a** The pull-down assay with the recombinant human kallistatin. The membrane proteins of NCI-H446 cell were pulled down by recombinant human kallistatin and subjected to immunoblotting with antibody anti-integrinβ3 (*upper panel*) and anti-kallistatin, respectively (*lower panel*). The membrane proteins incubated with Ni–NTA alone were used as control. **b** Immunoprecipitation of membrane proteins of NCI-H446 cell with anti-kallistatin antibody followed by immunoblotting against integrin β3 and kallistatin, respectively. The extracts of NCI-H446 cells treated without kallistatin were immunoprecipitatedas control. Co-immunoprecipitation sample without treated with Protein A-Sepharose were used to immunoblotting as input. **c** Immunoprecipitation of membrane proteins of NCI-H446 cell with anti-Integrin β3 antibody followed by immunoblotting against integrin β3 and kallistatin. The extracts of NCI-H446 cells treated without kallistatin were immunoprecipitated as control. Co-immunoprecipitation samples without treated with Protein A-Sepharose were used to immunoblotting as input

We selected NCI-H446 cells as target cells to identify kallistatin-binding partners because they were sensitive to kallistatin as assayed on small cell lung cancer cell viability and proliferation (data not shown). NCI-H446 cell sensitivity to kallistatin suggested that they might express potential receptor(s) for kallistatin. To identify kallistatin-binding proteins, kallistatin-Ni–NTA affinity beads were prepared. Pull-down assay revealed that the His-kallistatin protein interacted with integrin β3 in the eluted fraction (Fig. 1a, upper panel) as well as purified recombinant kallistatin (Fig. 1a, lower panel).

To identify kallistatin-binding proteins, NCI-H446 cells were treated with kallistatin. Eight hours after treatment, cell lysates were immunoprecipitated with anti-kallistatin antibody, anti-integrin β3 or anti-rabbit IgG antibody, and the resulting immunoprecipitates were separated by SDS-PAGE and immunoblotted. Immunoblotting using anti-kallistatin antibody showed that kallistatin co-precipitated with integrin β3 (Fig. 1b). To further confirm the interaction between integrin β3 and kallistatin, integrin β3 antibody was used for immunoprecipitation followed by immunoblotting analysis, thus showing that integrin β3 co-precipitated with kallistatin (Fig. 1c). Together, these results indicated that kallistatin specifically bound to integrin β3 in NCI-H446 cells.

### Effect of kallistatin on NCI-H446 and CHO cell viability

**Fig. 2:**
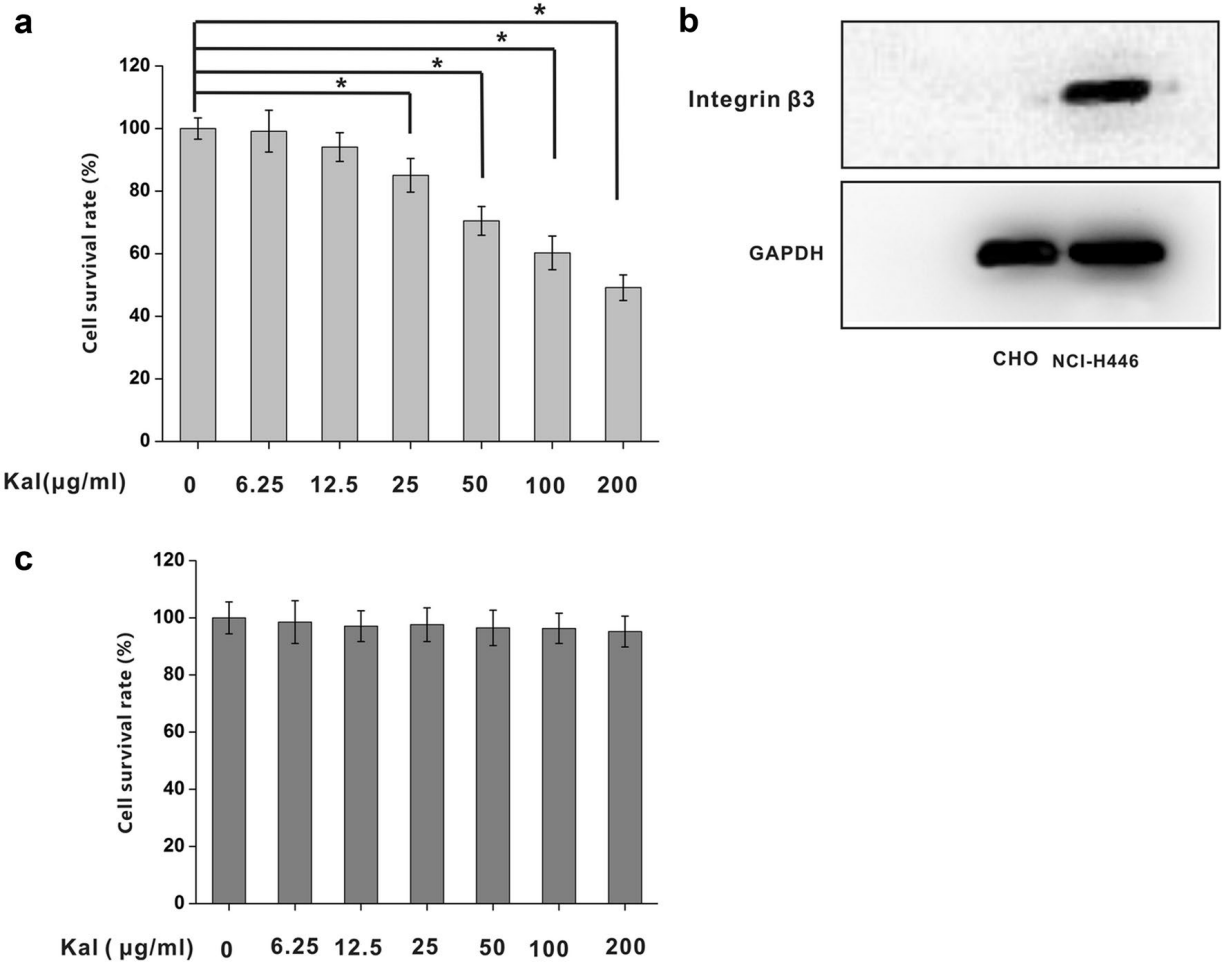
The effect of kallistatin on viability of NCI-H446 and CHO cells. **a** The effects of kallistatin on the viability of NCI-H446 cells in a dose dependent manner. N = 6 × 3 for each group. *P < 0.05. **b** Detect integrin expression in NCI-H446 and CHO cells by western blot. **c** Effect of kallistatin on cell viability in CHO cells which is lack of integrin β3 genes as a negative control. N = 6 × 3 for each group

Kallistatin inhibited the viability of small cell lung cancer NCI-H446 cells in a dose-dependent manner in vitro (Fig. 2a). While the viability of NCI-H446 cells was reduced but with no statistical significance (*P* > 0.05) by kallistatin at low does (6.25–12.5 μg/ml), it was reduced significantly inhibited in medium-dose (25–50 μg/ml) (*P* < 0.05) and high-dose (100–200 μg/ml) groups (*P* < 0.05), respectively. Non-integrin β3–expressing cells were used as controls, and CHO cell viability was not suppressed by kallistatin (Fig. 2b, c).

### Inhibition of integrin β3 attenuates the effects of kallistatin on NCI-H446 cell viability

**Fig. 3:**
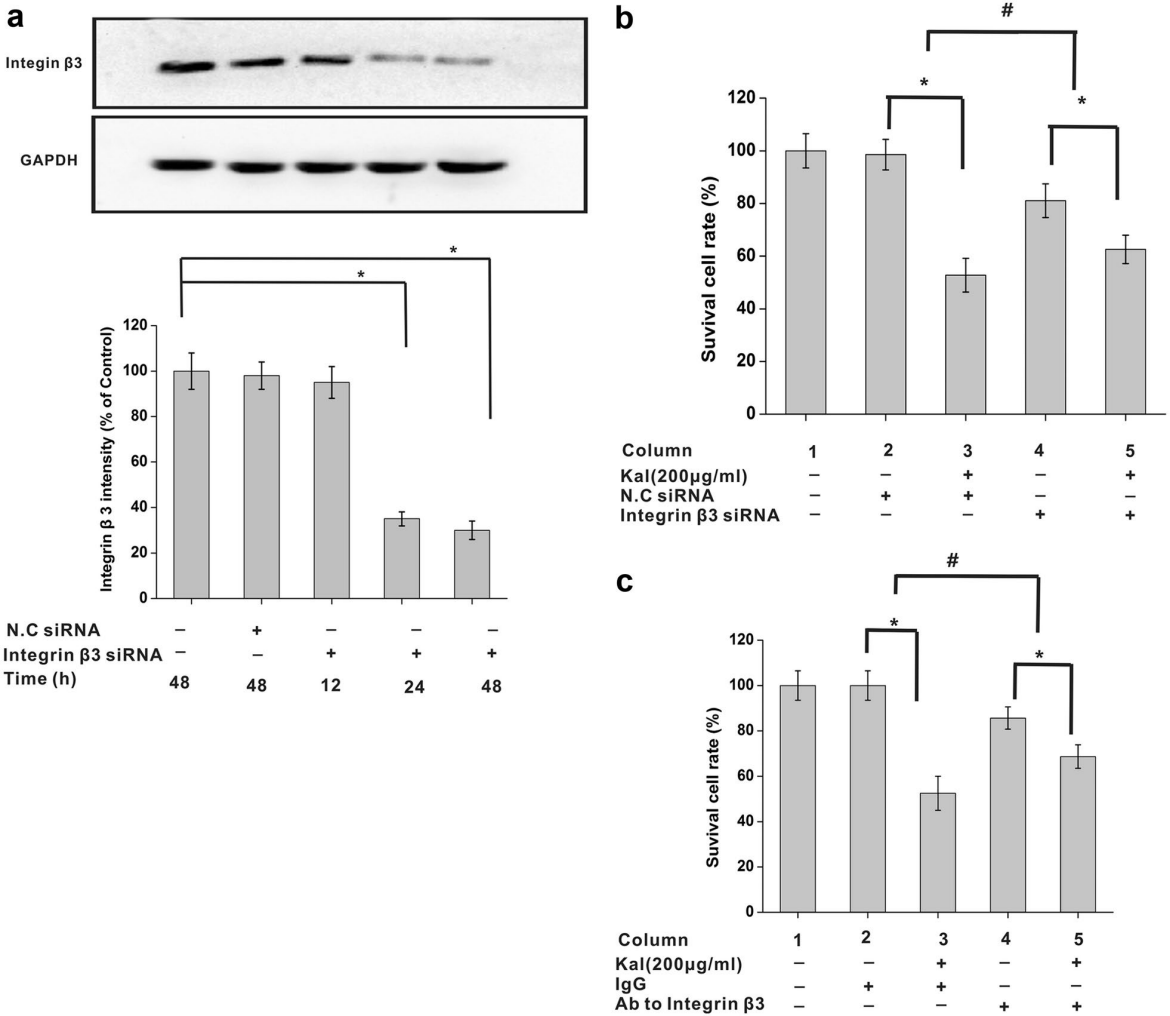
Integrin β3 is involved in the effects of kallistatin on viability of NCI-H446 cells. **a** Integrin β3 expression in NCI-H446 cells was downregulated by siRNA in a time-dependent manner that was analyzed by immunoblotting (N = 3). Housekeeping proteins GAPDH are useful as loading controls for immunoblotting and protein normalization. The data are expressed as mean ± SD from three independent experiments. *P < 0.05. **b** Inhibition effect of kallistatin on the viability of NCI-H446 cells was attenuated by treatment of siRNA. N = 6×3 for each group. N.C siRNA was used as negative control, respectively. *P < 0.05, ^#^P < 0.05, ^#^ comparison of the differences between column 2 and 3 and between column 4 and 5. **b** Inhibition effect of kallistatin on the viability of NCI-H446 cells was attenuated by antibody against integrin β3. N = 6 × 3 for each group. Mouse IgG were used as negative control. *P < 0.05, ^#^P < 0.05, ^#^ comparison of the differences between column 2 and 3 and between column 4 and 5

To further investigate the functional relationship between kallistatin and integrin β3, we analyzed the viability of NCI-H446 cells exposed to kallistatin (200 μg/ml), with different expression levels of integrin β3. The integrin β3 protein was downregulated by the siRNA method (Fig. 3a) and blocked by antibody against human integrin β3, respectively. While NCI-H446 cell viability was markedly inhibited by kallistatin treatment, the inhibitory effect was attenuated by either integrin β3 silencing by siRNA (Fig. 3b) or neutralization by specific antibodies (Fig. 3c). These findings indicated that integrin β3 was involved in the effect of kallistatin on NCI-H446 cell viability.

### Inhibition of integrin β3 attenuates the inhibitory effect of kallistatin on NCI-H446 cell proliferation

**Fig. 4:**
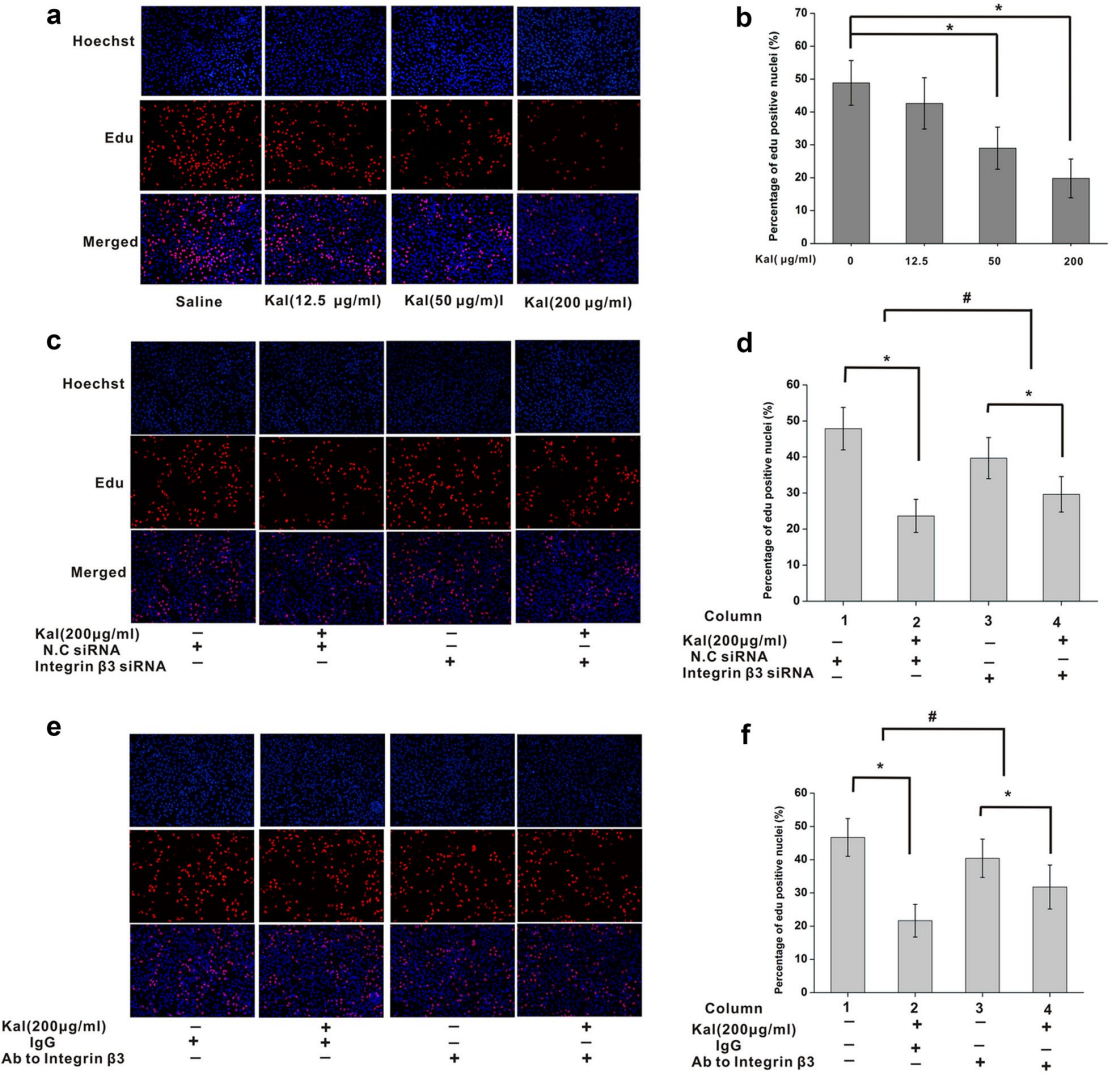
Integrin β3 is involved in the inhibition effect of kallistatin on proliferation of NCI-H446 cells. **a** Proliferation of NCI-H446 cells was inhibited in presence of kallistatin. Proliferating NCI-H446 cells were labeled with EdU (*red*). Cell nuclei were stained with Hoechst 33,342 (*blue*). **b** The percentage of EdU-positive NCI-H446 cells was quantified. Kallistatin inhibited the proliferation of NCI-H446 cells in a dose-dependent manner. Data were presented as mean ± SD (N = 6). *P < 0.05. **c** Down-regulation of integrin β3 by siRNA attenuated the proliferation inhibition of NCI-H446 cells at 48 h post kallistatin treatment as measured by EdU assay. **d** The percentage of EdU-positive NCI-H446 cells was quantified. Data were presented as mean ± SD (N = 6). N.C siRNA was used as negative control. *P < 0.05, ^#^P < 0.05, ^#^ comparison of the differences between column 1 and 2 and between column 3 and 4. **e** Blockage of integrin β3 with antibody (anti-integrin β3) attenuated the viability inhibition of NCI-H446 cells at 48 h post kallistatin treatment as measured by EdU assay. **f** The percentage of EdU-positive NCI-H446 cells was quantified. Data were presented as mean ± SD (N = 6). Mouse IgG was used as negative control. *P < 0.05, ^#^P < 0.05, ^#^ comparison of the differences between column 1 and 2 and between column 3 and 4

We further assessed the effect of kallistatin on NCI-H446 cell proliferation. The thymidine analogue EdU was present the entire duration of cell culture, thereby cumulatively labeling all nuclei undergoing or having undergone cell division, thus serving as a measure of the total number of daughter cells being generated in the culture. As shown in Fig. 4a and b, a significant difference in medium-(29.01 ± 6.40%) and high-(19.80 ± 5.96%) dose was detected in kallistatin groups, compared with the saline group (48.86 ± 6.82%) (*P* < 0.05), indicating that kallistatin inhibited NCI-H446 cell proliferation in a dose-dependent manner. No significant difference between low-dose (42.68 ± 7.83%) and saline groups was found.

If integrin β3 is involved in inhibiting NCI-H446 cell proliferation under kallistatin exposure, such inhibition could be reverted by down-regulating or blocking integrin β3. Comparing kallistatin and NC siRNA groups, indeed, more EdU positive nuclei were found in the kallistatin groups after integrin β3 silencing by siRNA (Fig. 4c, d). Importantly, the effect of integrin β3 blockage on kallistatin induced inhibition of cell proliferation was in full agreement with integrin β3 down-regulation findings. A significant decrease in cell proliferation was also observed when kallistatin was applied together with the mouse anti-human integrin β3 monoclonal antibody (Fig. 4e, f). There were significant differences between the scramble siRNA and integrin β3 siRNA groups (Fig. 4d). Therefore, down-regulation of integrin β3 markedly attenuated the inhibitory effects of kallistatin on NCI-H446 proliferation (Fig. 4f). Similar results were observed after integrin β3 was blocked by the mouse anti-human integrin β3 monoclonal antibody (Fig. 4f). These data demonstrated that kallistatin inhibited cell proliferation in an integrin β3 associated manner.

### Integrin β3 inhibition attenuates the suppressive effects of kallistatin on NCI-H446 cell migration

**Fig. 5:**
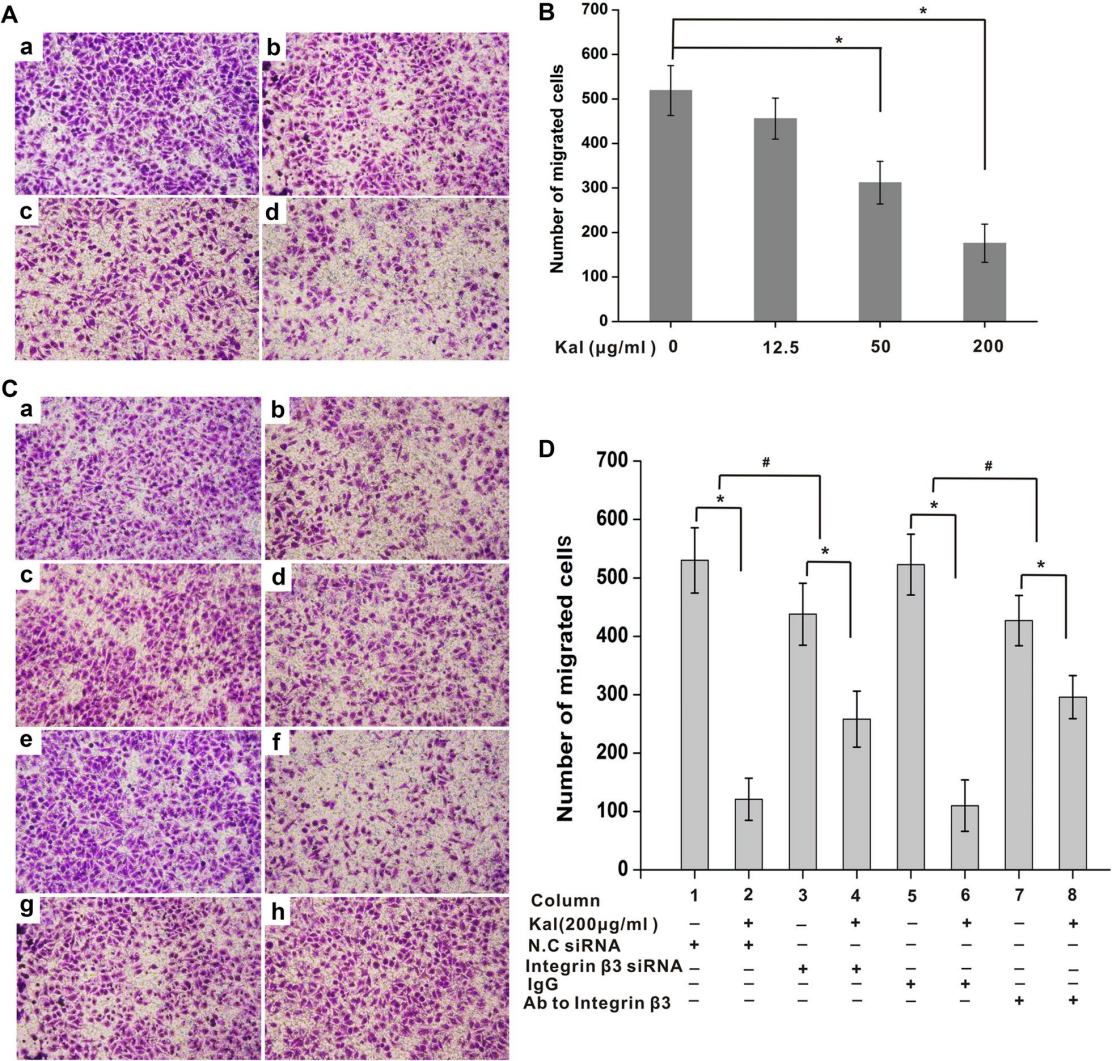
Reduction effects of kallistatin on NCI-H446 cells migration can be diminished by inhibition of integrin β3. **A** Optical microscopy imaging of the cells in experimental transwells. The cells have migrated from upper chamber to underside of the membrane between the two chambers after treatment with kallistatin. *a* saline; *b* 12.5 μg/ml kallistatin; *c* 50 μg/ml kallistatin; *d* 200 μg/ml kallistatin. **B** Statistic analysis for the migrated cell numbers. **C** Either down-regulation of integrin β3 by siRNA or blockage of integrin β3 with antibody (anti-integrin β3) attenuated the reduction effects of kallistatin on NCI-H446 cell migration. *a* Transfected with negative control siRNA and treated with saline; *b* transfected with negative control siRNA and treated with 200 μg/ml kallistatin; *c* transfected with integrin β3 siRNA and treated with saline; *d* transfected with integrin β3 siRNA and treated with 200 μg/ml kallistatin; *e* treated with mouse IgG and saline; *f* treated with mouse IgG and 200 μg/ml kallistatin; *g* treated with integrin β3 antibody and saline; *h* treated with integrin β3 antibody and 200 μg/ml kallistatin. **D** Statistic analysis for the migrated cell numbers. Data were presented as mean ± SD (N = 6). *P < 0.05.^#^P < 0.05,^#^ comparison of the differences between column 1 and 2 and between column 3 and 4, and the differences between column 5 and 6 and between 7 and 8

The effect of kallistatin on the chemotactic motility of NCI-H446 cells was measured by the Transwell chamber assay. Interestingly, kallistatin reduced NCI-H446 cell migration, in a concentration-dependent manner (Fig. 5A, B).

To further assess the importance of integrin β3 in kallistatin induced suppressive effects on cell migration, integrin β3 expression was reduced by siRNA. As shown in Fig. 5C, integrin β3 down-regulation abolished partly but significantly kallistatin-inhibited cell migration. while the negative control siRNA had no overt effects. Similarly kallistatin-inhibited cell migration was abolished partly by treatment with the mouse anti-human integrin β3 monoclonal antibody (Fig. 5D).

### Kallistatin suppresses integrin β3-FAK-Src signaling

**Fig. 6:**
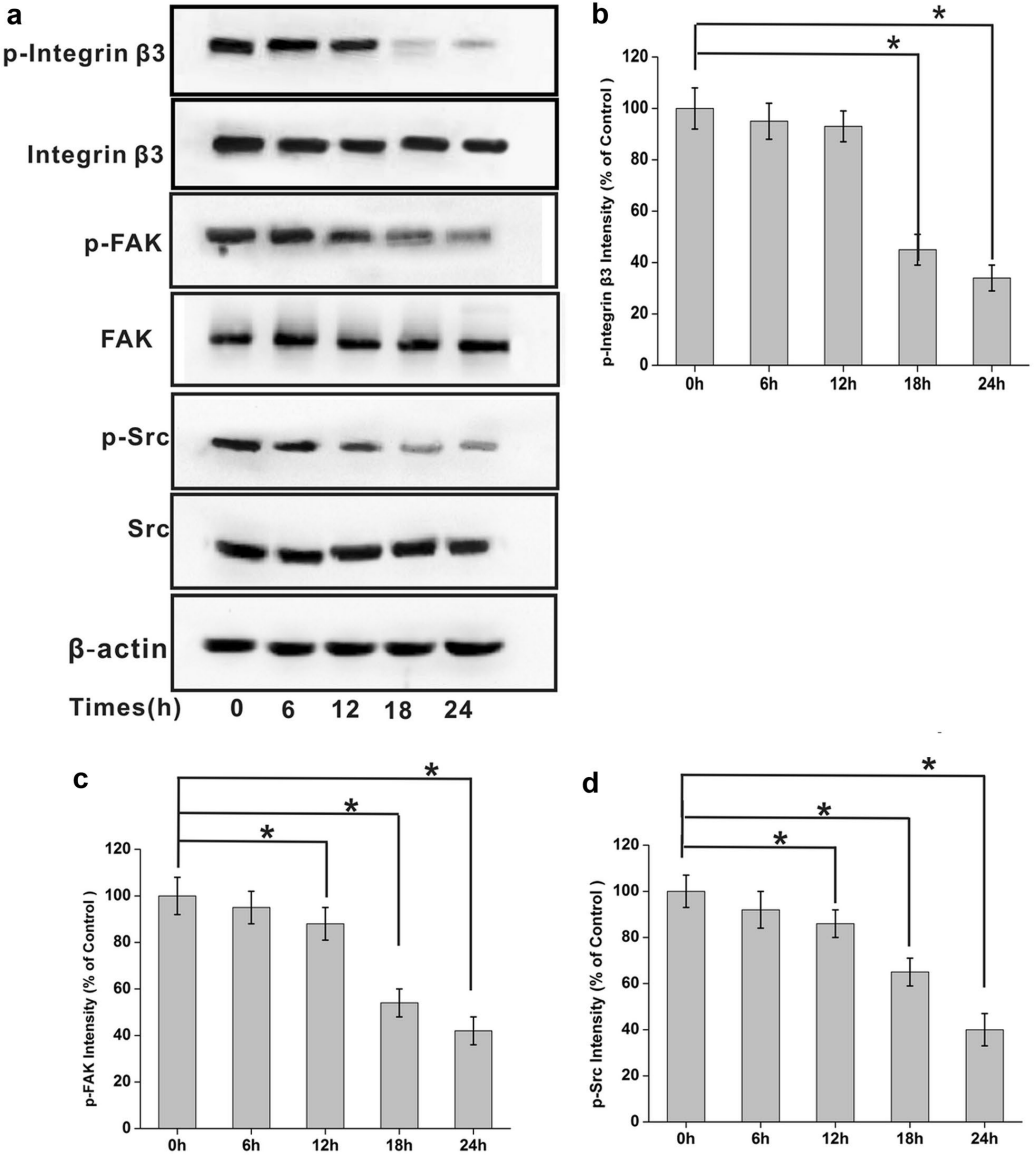
Kallistatin inhibits phosphorylation along the integrin signaling pathway. **a** 200 μg total cellular protein of each sample was used for immunoblotting analysis. Phosphorylation of integrin β3, FAK and Src was inhibited in a time-dependent manner by treatment of kallistatin, while each of their total protein levels showed undetectable change. (N = 3). The protein levels were normalized with β-actin. **b**–**d** The relative levels of phosphorylated integrin β3, phosphorylated FAK and phosphorylated Src proteins were calculated and plotted. The data are expressed as mean ± SD from three independent experiments (N = 3). *P < 0.05

Based on the above results, we sought to unveil the signaling pathway involved in the interaction of kallistatin and integrin β3. NCI-H446 cells were treated with kallistatin (100 μg/ml) for different times (0, 6, 12, 24, and 48 h) before lysis. The lysates were used for immunoblotting to analyze phosphorylation levels of integrin β3-FAK-Src signaling pathway effectors. Interestingly, phosphorylation of integrin β3, FAK and Src was inhibited in a time-dependent manner, while total levels of these proteins were virtually unchanged (Fig. 6). It is well known that integrin β3 alters cellular behaviors, including cell shape and mobility, through recruitment and activation of FAK and Src that form a dual kinase complex. Phosphorylation levels of integrin β3, FAK and Src, respectively, in NCI-H446 cells were markedly decreased after treatment with kallistatin, indicating that kallistatin acted as an antagonist of integrin β3-FAK-Src signaling, by interaction with integrin β3 in NCI-H446 cells.

### Kallistatin suppresses integrin β3-mediated PI3-K/AKT signaling

**Fig. 7:**
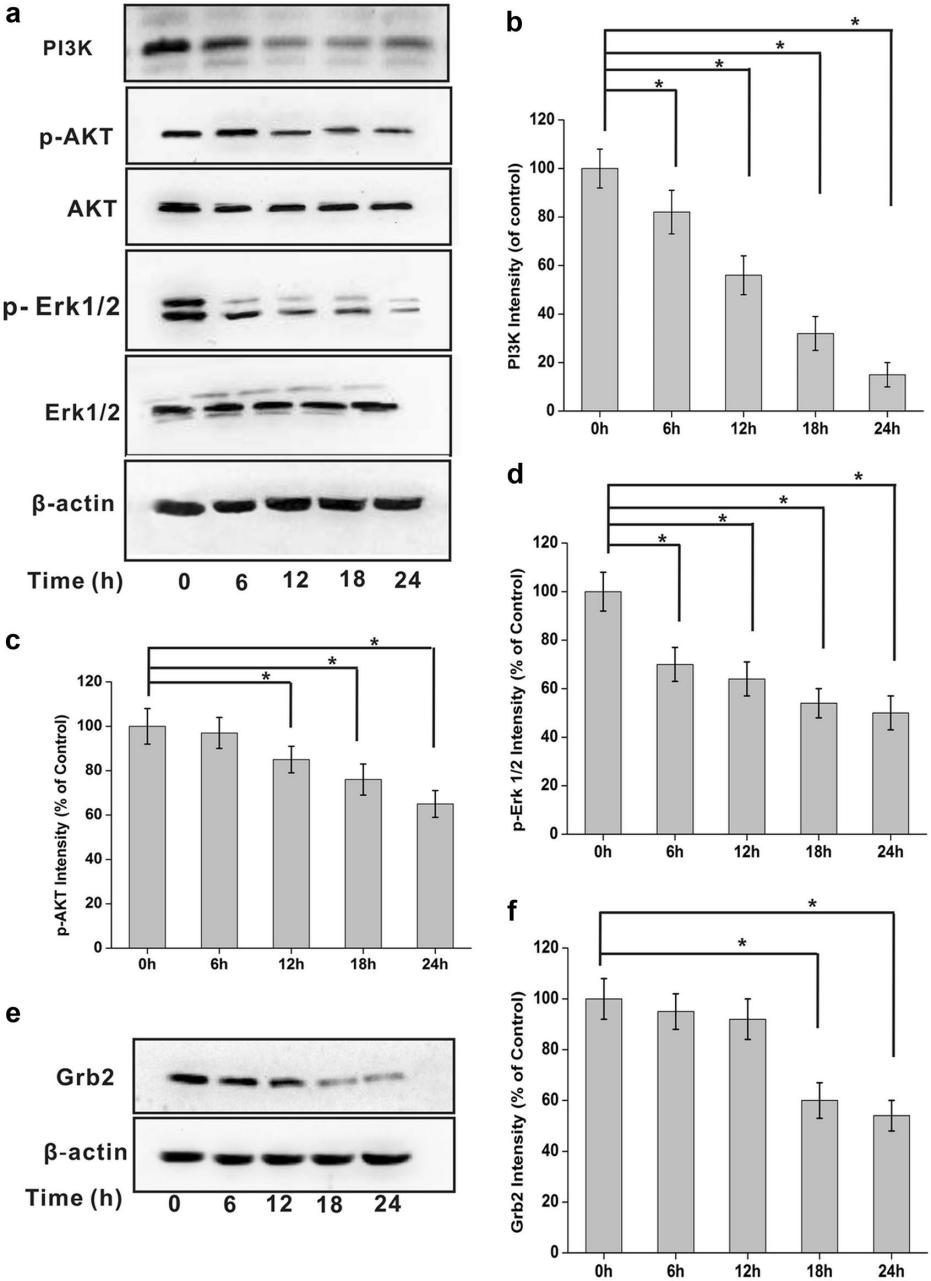
Kallistatin inhibits integrin β3-mediated PI3-K/AKT pathwaysignaling. **a** and **e** Using 200 μg total cellular proteins from each of the samples for immunoblotting analysis. The protein levels were normalized with β-actin. **a** Phosphorylation of AKT Erk1/2 and PI3 K were inhibited in a time-dependent manner without detectable change of their total AKT and Erk1/2 protein levels after treated with kallistatin (N = 3). (**b**–**d**) The relative levels of PI3 K, phosphorylated AKT and phosphorylated Erk1/2 proteins were calculated and plotted. The data are expressed as mean ± SD from three independent experiments (N = 3). *P < 0.05. **f** The relative levels of Grb2 proteins were calculated and plotted. The data are expressed as mean ± SD from three independent experiments (N = 3). *P < 0.05

Integrin-mediated FAK/Src signaling allows downstream activation of many pathways, including PI3-K/AKT and Erk/MAPK signaling, which promote cell survival and proliferation. NCI-H446 cells were treated with kallistatin for various times (0, 6, 12, 24, and 48 h). Then, cells were lysed for immunoblotting. Interestingly, phosphorylated AKT and Erk1/2 levels and total PI3 kinase amounts were reduced, while no change in total AKT and Erk levels were detectable after kallistatin treatment (Fig. 7a, b).

Grb2 is a key adaptor protein that maintains the ERK activity. We found that kallistatin decreased Grb2 expression (Fig. 7c, d), and reduced Erk1/2 phosphorylation, in a time-dependent manner (Fig. 7a, b), suggesting that suppression of Grb2 might be one of the molecular mechanisms behind kallistatin’s effects.

### Kallistatin induces NCI-H446 apoptosis

**Fig. 8:**
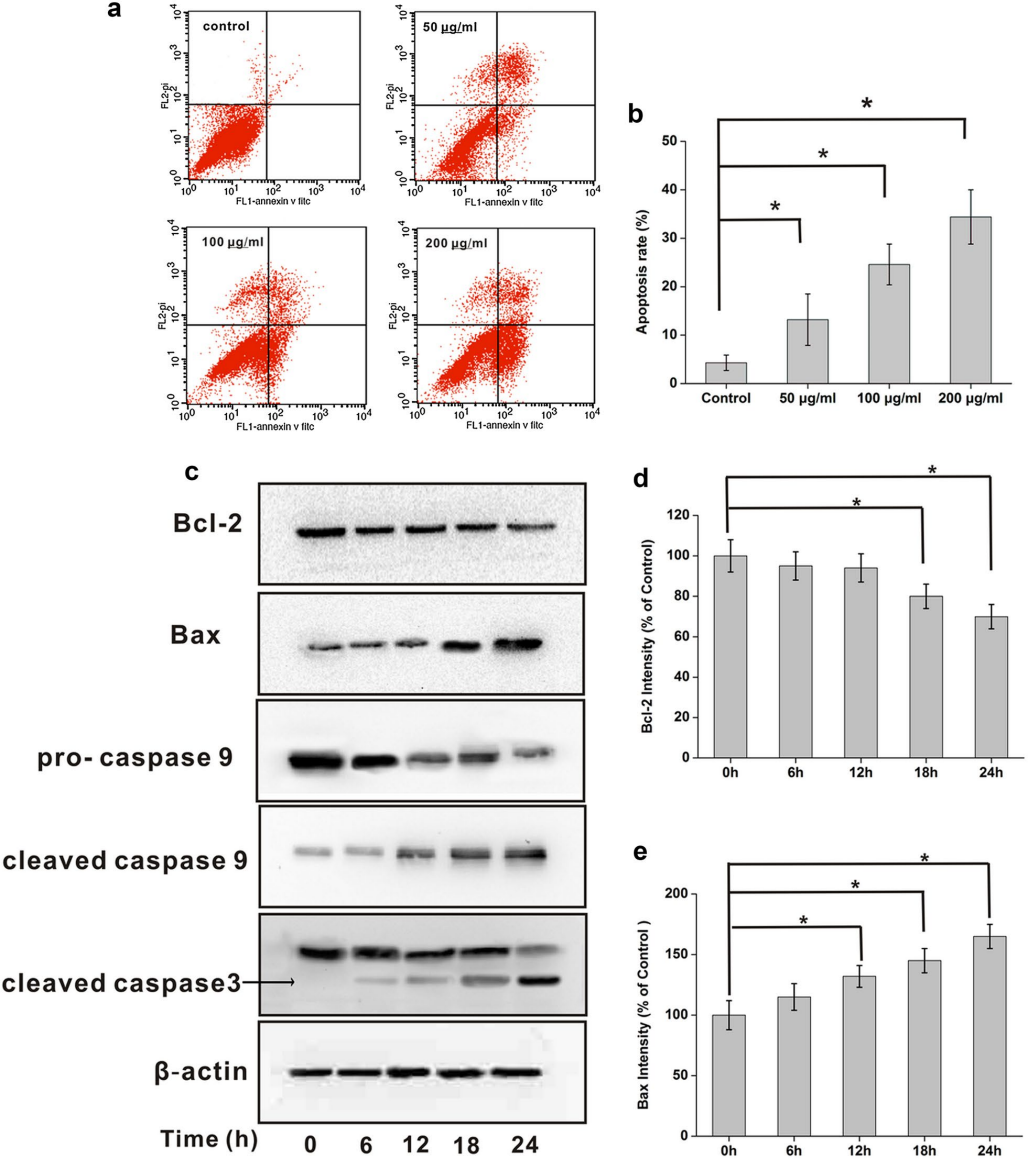
Kallistatin induces NCI-H446 cells apoptosis. **a** The cells were collected and stained with annexin V-FITC/PI (1: PBS; 2: 50 µg/ml kallistatin; 3: 100 µg/ml kallistatin; 4: 200 µg/ml kallistatin); **b** the quantitative results of flow cytometry analysis. *P < 0.05, N = 3. **c** Immunoblotting were performed using 200 μg total proteins from each sample. Relative levels of Bcl-2 and pro-caspase 9 were decreased, while Bax, cleaved caspase 9 and cleaved caspase-3 were increased. The protein levels were normalized with β-actin. **d**, **e** The relative Bcl-2, Bax levels were calculated and plotted. The data are expressed as mean ± SD from three independent experiments (N = 3 each). *P < 0.05

Based on the effects on NCI-H446 cell viability and proliferation, kallistatin at doses of 50, 100 and 200 μg/ml was assessed for its inhibitory effects on cell apoptosis by flow cytometry; apoptosis rates were 13.4, 24.2, and 34.5% respectively. These results showed that kallistatin induced apoptosis in a concentration dependent manner (Fig. 8a, b).

**Fig. 9:**
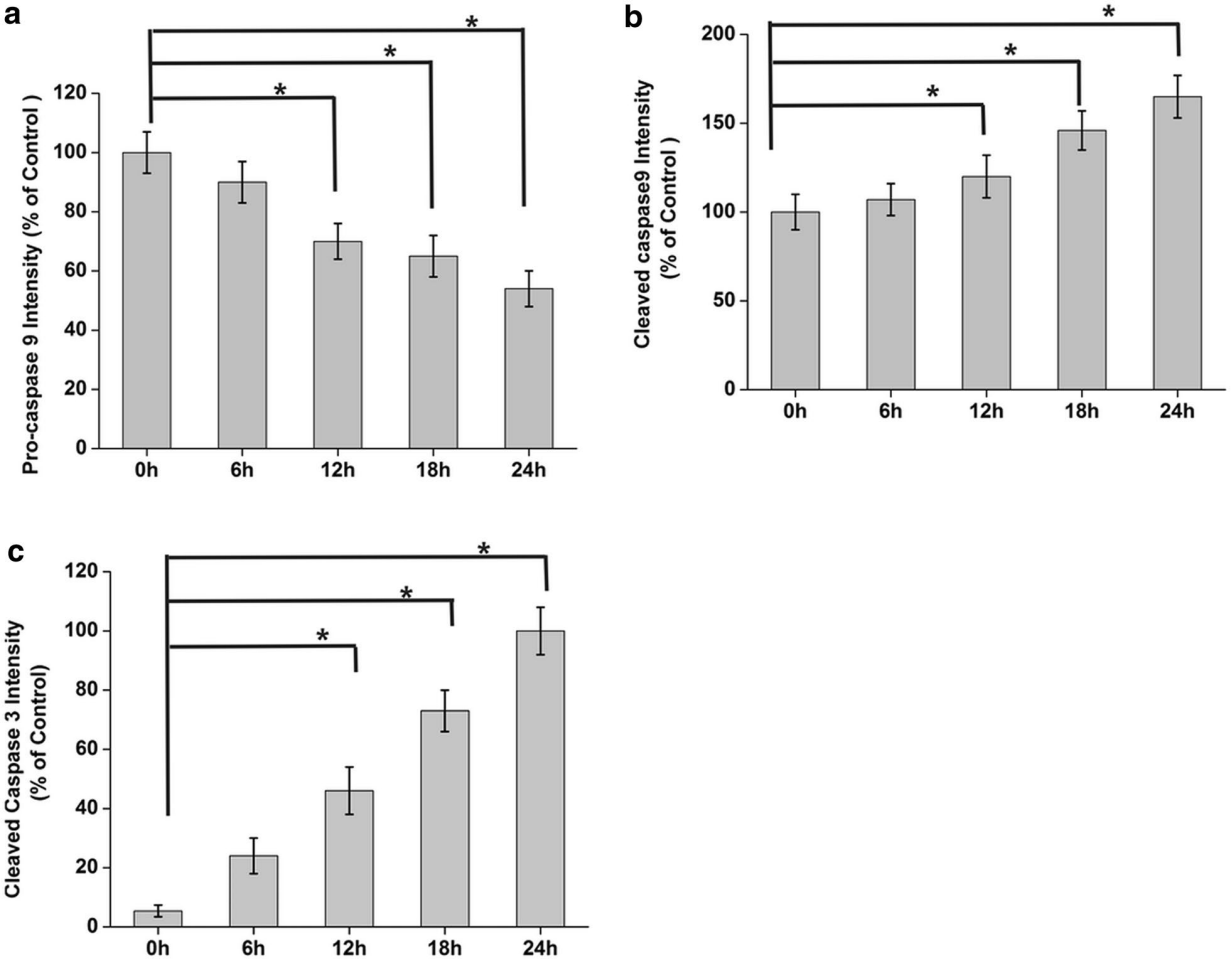
Statistical analysis of apoptosis related molecules induced by kallistatin. **a**–**c** The relative pro-caspase 9, cleaved caspase 9 and cleaved caspase 3 levels were calculated and plotted. The data are expressed as mean ± SD from three independent experiments (N = 3 each). *P < 0.05

NCI-H446 cells were treated with kallistatin (100 μg/ml) for various times (0, 6, 12, 24, 48 h), and cell lysates were assessed by immunoblotting. Interestingly, relative levels of Bcl-2 and pro-caspase 9 were decreased, while Bax, cleaved caspase-9 and caspase-3 amounts were increased (Figs. 8c−e, 9a–c).

## Discussion

**Fig. 10:**
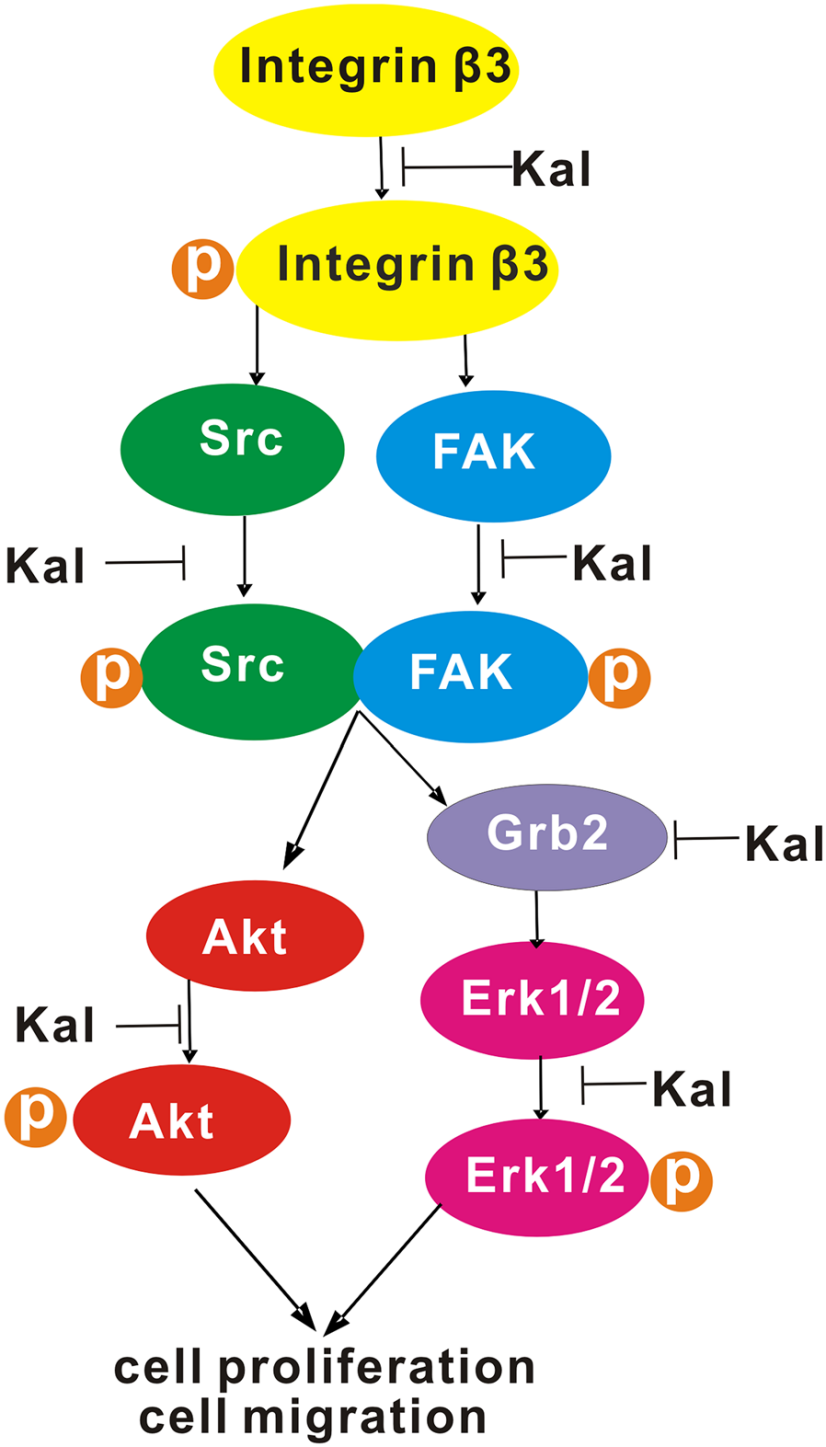
The proposed effect sites of kallistatin through integrin signaling pathway. Kallistatin inhibited the both of PI3 K/AKT and Erk/MAPK resulted in a downregulation of Bcl-2 expression for the anti-apoptotic homologs, and an upregulation for the pro-apoptotic homologs, and inhinited the phosphorylation integrin β3 and suppresses the expression of Grb2 for the cell migration

The present study identified integrin β3 as a kallistatin binding protein in NCI-H446 cells, using pull-down assays, immunoprecipitation, and immunoblotting. Indeed, kallistatin bound directly to integrin β3, and inhibited integrin β3 phosphorylation that is required for the outside-in signaling cascade, thereby antagonizing β3-induced phosphorylation of FAK and Src, and suppressing AKT and Erk signaling. A schematic depicting the effect of kallistatin on integrin β3 signaling is shown in Fig. 10.

Increasing evidence suggests that specific integrin signals enable cancer cells to survive, proliferate and migrate during progression from tumor growth to metastasis. Integrin signals can also originate inside cells and affect receptor affinity, thereby controlling ligand binding, initiating a cross talk with other receptors, and altering cell adhesion and proliferation. In multiple tumor types, including glioma, breast cancer, and melanoma, integrin β3 expression promotes invasion and metastasis [29]. On the other hand, more and more studies indicate that kallistatin exerts pleiotropic effects in not only inhibiting tumor angiogenesis and inflammation, but also reducing tumor growth and metastasis directly. For example, local delivery of the human kallistatin gene significantly reduces tumor growth and angiogenesis in a NCI-H446 subcutaneous xenograft tumor model [30]. Systemic administration of lentiviral vectors encoding kallistatin inhibited the growth of metastatic lung tumors and prolonged survival in tumor-bearing mice [21]. Kallistatin suppresses tumor growth and angiogenesis in nude mice by antagonizing VEGF-mediated cell proliferation, migration, and invasion of endothelial cells [17]. A recent study revealed a novel mechanism mediated by kallistatin via antagonization of canonical Wnt signaling, leading to a retardation of tumor progression [20]. Since kallistatin inhibits the growth of NCI-H446 cells directly [23], we assessed whether kallistatin also participates in signaling via other receptors. This is the first study demonstrating that kallistatin inhibits the integrin β3 signaling cascade in lung cancer cells.

Multiple roles for FAK signaling have been demonstrated in the early stages of mammary carcinoma lung metastasis [31]. Increased phosphorylation of FAK in lung cancer is closely correlated with nodal involvement and short disease-free survival time [32]. In an experimental model of metastatic mammary carcinoma, lung metastasis formation was prevented with a dominant-negative FAK inhibitor expressed one day before tumor cell injection [31], suggesting this pathway might serve as a therapeutic target in lung tumorigenesis. Kallistatin treatment in NCI-H446 cells prevented FAK phosphorylation and FAK-Src complex activation, thereby reducing both cell proliferation and motility. These findings provided an experimental basis for targeting invasion and metastasis with kallistatin as an FAK inhibitor.

Targeting FAK/Src pathway kinases in solid tumors is considered an attractive therapeutic approach via inhibition of Src family kinases. Multiple studies have definitely identified Src as a key player in tumor progression, as it can provide oncogenic signals for cell survival, invasion and metastasis [33]. Indeed, Src inhibition in cancer cells results in E-cadherin upregulation, enhanced cell–cell adhesiveness, and markedly reduced metastatic rate [34]. Src inhibition in human colorectal cancer cells prevents Src-dependent β-catenin phosphorylation and β-catenin-related transcriptional activity, thereby reducing both cell proliferation and motility [35]. As shown above, kallistatin inhibited both FAK and Src phosphorylation, and repressed downstream signaling cascades such as ERK/MAPK and PI3K/AKT signaling pathways that control tumor growth as well as other cellular processes such as cell motility and adhesion, suggesting a potential clinical usefulness of kallistatin.

It is well known that PI3K plays an important role in integrin β3 regulation. Therefore, the effects of kallistatin on the phosphorylation of the PI3K downstream target AKT, which is essential for numerous cell survival functions, were assessed. AKT plays a critical role in controlling the balance between cell survival and apoptosis, and phosphorylation is required for its activation. Once activated, AKT inactivates proapoptotic proteins, including the Bcl-2 family member Bad and caspase-9, as well as cell cycle regulatory molecules. The ERK/MAPK pathway has also been identified as an important cell survival-promoting pathway. Ras activation initiates a multistep phosphorylation cascade that leads to the activation of MAPKs. Meanwhile, MAPKs and ERK1 have been linked to cell proliferation, survival, and migration. As shown above, treatment with kallistatin not only resulted in abrogated AKT activation, but also reduced Grb2 levels and induced ERK1/2 phosphorylation. These important functions make Grb2 a logical therapeutic target for strategies designed to prevent the spread of solid tumors via local invasion and metastasis. Inhibition of both PI3K/AKT and Erk/MAPK resulted in Bcl-2 downregulation, and an upregulation of pro-apoptotic homologs, as well as caspase-9 and caspase-3 activation [36]. Kallistatin treatment of NCI-H446 decreased the relative levels of Bcl-2 and pro-caspase 9, while Bax, cleaved caspase-9 and caspase-3 were increased.

The molecular pathways involved in survival and replication of cancer cells are very complex, and interfering with only single steps may often be insufficient for therapy. Integrin β3 signaling represents an example of such complexity. Although its primary function is thought to be coordination of cell-matrix communication to affect intracellular signaling cascades, integrin β3 is capable of triggering anchorage-independent cell survival and tumor metastasis in the absence of ligand binding. These events in turn activate downstream signaling pathways, including the MAPK and PI3K/AKT pathways. Cancer cells have an inherent ability to harness diverse growth factor signaling pathways for growth advantage and survival; blocking only one molecular target allows others to act in salvage or escape mechanisms for cancer cells. Preclinical and clinical evidence of synergistic antitumor activity achievable by combining targeted agents that block multiple signaling pathways has recently emerged [37, 38]. Agents that intrinsically target various signaling pathway molecules may represent a hot topic in the field of cancer research. The current study revealed that kallistatin targets multiple signaling pathways by antagonizing canonical integrin β3 signaling, leading to reduced lung cancer cell proliferation, survival and migration. Integrin β3 is upregulated in both tumors and angiogenic endothelial cells, making them targets of both angiogenic activators and inhibitors. Function-blocking monoclonal antibodies, such as LM609 and etaracizumab were among the first integrin antagonists developed, with considerable anti-angiogenic activity in preclinical models. Several endogenous angiogenic inhibitors, including endostatin and tumstatin, seem to exert their function by blocking integrins. The current study showed that kallistatin can bind to integrin β3 and inhibit the integrin β3 signaling pathway. These findings suggested that kallistatin, as an integrin β3 inhibitor, can be used for anti-tumor therapies and provide essential insights for the biological role of kallistatin in tumor growth and progression.

## Conclusion

In summary, this study showed that blockage of integrin β3 signaling by kallistatin negatively affects lung cancer cell proliferation, survival and migration. These results contribute to further understanding of the molecular mechanisms by which kallistatin targets multiple integrin-mediated processes in lung cancer.

## Abbreviations

ECM: cell-extracellular matrix
PTKs: protein-tyrosine kinases
AKT: protein kinase B
FAK: focal adhesion kinase
Grb: growth factor receptor-bound protein
eNOS: endothelial nitric oxide synthase
LRP6: low-density lipoprotein receptor-related protein 6
KLF-4: Kruppel- like factor 4
Erk: extracellular signal-regulated kinase
MTT: 3-(4,5-dimethylthiazol-2-yl)-2,5-diphenyl-tetrazolium bromide
CHO: Chinese hamster ovary cell
EdU: 5-ethynyl-2-deoxyuridine
MAPK: Ras–mitogen-activated protein kinase
PI3K: phosphoinositide 3-kinase
PBS: phosphate-buffered saline
